# Metabolic disrupting chemicals in the intestine: the need for biologically relevant models: Zebrafish: what can we learn from this small environment‐sensitive fish?

**DOI:** 10.1002/2211-5463.13878

**Published:** 2024-09-01

**Authors:** Chedi Erradhouani, Sylvie Bortoli, Selim Aït‐Aïssa, Xavier Coumoul, François Brion

**Affiliations:** ^1^ Ecotoxicologie des Substances et des Milieux INERIS Verneuil‐en‐Halatte France; ^2^ Université Paris Cité France; ^3^ Inserm UMR‐S 1124 Paris France

**Keywords:** CYP3A4, endocrine disruptors, fish models, gut metabolism, *in vivo* models, zebrafish

## Abstract

Although the concept of endocrine disruptors first appeared almost 30 years ago, the relatively recent involvement of these substances in the etiology of metabolic pathologies (obesity, diabetes, hepatic steatosis, etc.) has given rise to the concept of Metabolic Disrupting Chemicals (MDCs). Organs such as the liver and adipose tissue have been well studied in the context of metabolic disruption by these substances. The intestine, however, has been relatively unexplored despite its close link with these organs. *In vivo* models are useful for the study of the effects of MDCs in the intestine and, in addition, allow investigations into interactions with the rest of the organism. In the latter respect, the zebrafish is an animal model which is used increasingly for the characterization of endocrine disruptors and its use as a model for assessing effects on the intestine will, no doubt, expand. This review aims to highlight the importance of the intestine in metabolism and present the zebrafish as a relevant alternative model for investigating the effect of pollutants in the intestine by focusing, in particular, on cytochrome P450 3A (CYP3A), one of the major molecular players in endogenous and MDCs metabolism in the gut.

## Graphical abstract

In this review, we aimed to study how the intestine may be disrupted by metabolic endocrine disruptors (MDCs) both in its own metabolism and in its relationships with other organs. Zebrafish shares numerous similarities with human gastrointestinal tract, representing an opportunity to study the complex interactions of MDCs at the intestinal level and the subsequent development of pathologies.

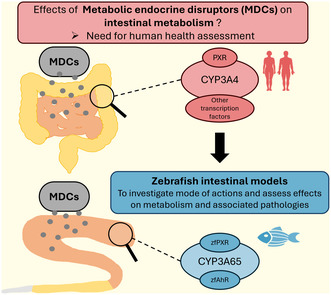

**Abbreviations**
AhR: aryl hydrocarbon receptor
BPA: bisphenol A
CAR: constitutive androstane receptor
CYPs: cytochromes P450
DEHP: diethylhexyl phthalate
EDCs: endocrine‐disrupting chemicals
GIT: gastrointestinal tract
GR: glucocorticoid receptor
HNF4α: hepatocyte nuclear factor 4 alpha
IBD: inflammatory bowel disease
LBD: ligand‐binding domain
MAFLD: metabolic dysfunction‐associated fatty liver disease
MDCs: metabolic disrupting chemicals
MetS: metabolic syndrome
PCB: polychlorinated biphenyl
POPs: persistent organic pollutants
PPARγ: peroxisome proliferator‐activated receptor gamma
PXR: pregnane X receptor
RXR: retinoid X receptor
TNFα: tumor necrosis factor alpha
WAT: white adipose tissue

video

## What are MDCs and what impact do they have on metabolic organs?

Of the 85 000 man‐made chemicals currently produced worldwide, 1000 of them are considered or suspected to be endocrine‐disrupting chemicals (EDCs) [1]. This is probably a significant underestimate since many substances are not well characterized, yet. Additionally, more than 9 million premature deaths, worldwide, are due to the contamination of air, water, food, and consumer goods. Chemical‐related diseases are, thus, one of the most important preventable forms of mortality [2]. Consequently, there is an urgent need for a strict assessment of compounds placed on the market and increased control of the substances released into the environment. Further attesting to this need is the fact that many persistent substances, although – having been banned for years, are still present in the environment in their native or metabolized forms [3].

### Metabolic endocrine‐disrupting chemicals (MDCs)

Recently, established links between EDCs and metabolic diseases like obesity have been reported increasingly. Obesity, a pandemic disease affecting 1 billion people worldwide in 2022 (more than three times the 1970s figures) [4], is linked to several metabolic diseases itself like type II diabetes, cardiovascular disorders, metabolic syndrome (MetS), metabolic dysfunction‐associated fatty liver disease (MAFLD), cancer and sterility [5, 6, 7].

Consequently, EDCs are investigated increasingly for their potential to increase the prevalence of metabolic diseases around the world [6]. Several publications established a link between EDCs and obesity, initially by serendipity [8]. In 2001, while studying the effect of perinatal exposure to bisphenol A (BPA) on the reproductive system in rats, Rubin *et al*. showed its implication in body weight gain [9]. Later, in 2005, Newbold *et al*. showed that exposure of newborns to diethylstilbestrol can lead to obesity in female mice [10]. In less than a decade, the links between childhood exposure to EDCs and obesity have become stronger which has led to the concept of obesogens [8]. These are “molecules that inappropriately regulate lipid metabolism and adipogenesis thus promoting obesity” [11]. In addition, these obesogens promote adipogenesis and weight gain which increases the retention of lipophilic pollutants thus perpetuating their harmful effects over time [12]. Furthermore, some obesogens may have transgenerational effects. For example, the fungicide tributyltin increases the weight of white adipose tissue, the size and the number of adipocytes and hepatic lipid accumulation in F1, F2, and F3 generation of mice following an exposure of the F0‐pregnant mice. This suggests that an early‐life obesogen exposure may have lasting effects which most likely involve epigenetic remodeling [13].

Four hundred and twenty‐two million people were known to be affected with diabetes in 2022 and this number is projected to increase in the coming years [14]. The causes of diabetes are, therefore, of particularly interest, especially in light of the fact that environmental factors are an increasingly important etiology for metabolic diseases. The link between EDCs, type II diabetes and MetS is attested by the adverse effects of EDCs on insulin secretion by the pancreatic β‐cells and by their peripheral actions, notably on muscle cells or adipocytes which are implicated in the development of insulin resistance. However, further research is needed to establish solid links between type 1 diabetes and EDCs [15]. As a result, the concept of Metabolic(‐endocrine) disrupting chemicals (MDCs) arose in 2015 [16]. This concept attempts to encompass, at first, both the notion of obesogens and chemicals potentially responsible for type 2 diabetes, MetS and liver abnormalities before extending to alterations of other metabolic organs such as the intestine.

### The principal organs investigated to determine MDCs effects

Historically, the effects of EDCs have been studied, mainly, in the context of reproduction and, more recently, neurodevelopment. The question of the impact of EDCs on other physiological functions such as immunity and metabolism has emerged more recently. Four organs are, currently, the main focus for research on the impacts of MDCs and metabolic diseases: the liver, adipose tissue, the pancreas, and the intestine, including possible dimorphic effects. Here, we provide a brief overview of the mechanisms and pathologies involved in the MDCs context for the liver, adipose tissue, and the pancreas before looking in more detail at the intestine.

#### Liver

Since the liver is responsible for the metabolism of many endogenous or exogenous molecules, it is an organ particularly well suited for the study of the response to xenobiotics, especially MDCs. A general increase in the incidence of obesity has occurred over the last decades and has been accompanied by an increase in the worldwide prevalence of MAFLD, an obesity co‐morbidity, from 25% in 1990–2006 to 38% in 2016–2019 [17]. Recently, MDCs have been identified, clearly, as risk factors in the etiology of this type of disease. By altering the activity of nuclear receptors and the aryl hydrocarbon receptor, MDCs modulate essential pathways for the regulation of lipid balance in the liver (such as carbohydrate and glucose metabolism), disrupt mitochondrial function and promote inflammatory processes in the liver. These effects lead to the development of MAFLD and potentially MASH (Metabolic dysfunction‐Associated Steatohepatitis) [18, 19, 20]. Additionally, once the storage capacity of white adipose tissue (WAT) has been attained, the liver begins to exhibit ectopic fat deposits that can lead, eventually, to the formation of non‐physiological lipid droplets by inflamed adipocytes [21].

#### Adipose tissue

White adipose tissue is a major target organ for MDCs. These compounds cause problems with calorie storage, especially since WAT is a preferred storage site for many persistent organic pollutants (POPs) that can be released over time [22]. Indeed, although adipose tissue has a storage function, it is also capable of releasing pollutants. This release of stored MDCs thus constitutes a long‐term internal exposure that can affect metabolism over time. For example, mice grafted with adipose tissue previously exposed to 2,3,7,8‐tetrachlorodibenzodioxin (TCDD) display signs of inflammation, gluconeogenesis and fibrosis both in the adipose tissue and the liver, 10 weeks after grafting [23].

#### Pancreas

The pancreas plays a fundamental role in regulating metabolism, notably through digestion and glycemia regulation. The disruption of its physiology by MDCs can lead to the appearance of serious metabolic pathologies such as diabetes. Although the responsibility of EDCs in the onset of diabetes mellitus, including the various types and subtypes of this complex disease, is strongly attested by an association with an exposure to several substances, a causal link needs to be clearly established. However, there is no longer any doubt that environmental contaminants such as BPA and TCDD are strong risk factors in the development of type 2 diabetes, notably through an alteration in the process of insulin secretion by β‐pancreatic cells [24, 25]. The link between diabetes and BPA can even be established through a metabolic reprogramming following gestational exposure. This can extend over several generations [26].

While the liver and adipose tissue have been relatively well studied, other organs, such as the pancreas and the intestine, have been less studied with respect to MDCs‐related metabolic pathologies.

#### Intestine

To get an overall view of the state of the researches conducted on the gut in the context of MDCs, we present here a review of articles published on the subject over the last 10 years. This compilation of publications was produced using the terms “(intestine AND “endocrine disruptors” AND metabolism)” between 2014 and 2024 in PubMed®, eliminating non‐English‐written papers or papers dealing with pharmacokinetics or methodology or not relevant to the human context. The publications are referenced according to the type of model used, the type of experiment (*in vitro*, *in vivo* or *in silico*), the EDC(s) investigated, the methods used, and the effects measured on the intestine. The results of this literature search are presented in Table 1.

**Table 1:** Summary of intestinal EDCs‐metabolism‐related studies published between 2014 and 2024.

Species	Experiment	Chemicals	Methods	Impact on the intestine	References
Amphibian and fish (various species)	*In vitro/in vivo*	BPA, triclosan, estradiol, heavy metal, etc.	Review	Dysbiosis	[143]
Fish (*Danio rerio*)	*In vivo*	BPA, SiO_2_ nanoparticles	Bioimaging of fluorescent nanoparticles	Accumulation in the intestine	[144]
		Bisphenols, pesticides, metals, phthalates, etc.	Review	Dysbiosis, genotoxic and mutagenic risk at the intestinal level	[145]
		Bisphenol AF	Intestinal histological analysis	No apparent alterations	[146]
Human	*In vitro*	Lilial	Intestinal barrier model (Caco‐2)	Not relevant for assessing lilial toxicity	[147]
		BPA	Review	Dysbiosis	[148]
		Monosodium glutamate	Intestinal cell line, ELISA	Impaired enteroendocrine glucagon‐like peptide‐1 hormone secretion	[149]
		Acetyl‐triethyl/tributyl‐citrates, phthalates	Intestinal transfected reporter human cell line (LS180), RT‐qPCR (e.g. *pxr*), cholesterol uptake assay	EDCs promote hypercholesterolemia through intestinal PXR	[95]
Human and rodent	*In vitro/in silico*	BPA, chlorpyrifos, diethylhexyl phthalate (DEHP), per‐ and polyfluoroalkyl substances	Review	Dysbiosis and intestine‐brain axis (neurotoxicity)	[40]
Human and rodent	*In vitro/in vivo*	Resveratrol	Review	Inhibits estrogen intestinal metabolism	[150]
		Bisphenols, pesticides, metals, phthalates, etc.	Review	EDCs‐related dysbiosis promotes metabolic diseases such as obesity	[37]
Human, rodent, fish	*In vitro/in vivo*	BPA analogs	Review	Impaired serotonin promoting obesogenic effects	[46]
		Various obesogens	Review	Dysbiosis and increased intestinal permeability	[151]
Mouse	*In vitro*	BPF and bisphenol S (BPS)	Fluorescence‐activated cell sorter analysis, cytokines measurements	Increased IL‐17 (po‐inflammatory) secretion in intestinal immune T cells	[152]
	*In vivo*	Polybrominated diphenyl ethers	Intestinal permeability assay with fluorescein isothiocyanate	No effect on intestinal permeability	[153]
		Di‐isononyl phthalate	Histology, RT‐qPCR for inflammation and gut integrity genes, cytokines and hormones measurements	Changes in histopathology, cytokines level, immune function and tight junctions	[51]
		BPA, DEHP, TCDD and polychlorinated biphenyl (PCB)	RT‐qPCR for xenobiotics processing intestinal genes (e.g. *cyp3a11*), lipoprotein‐lipase gene and inflammatory markers	Highly sensitive jejunum (for lipase and xenobiotics receptors)	[154]
		BPS	Cytokines, cholesterol and triacylglycerol measurements	Transgenerational intestinal inflammation	[52]
			RT‐qPCR (duodenum) of *glut2*, *apelin*	Impaired intestinal glucose absorption and glucose metabolism (increased risk of MetS)	[155]
		TCDD, PCB, BPA, DEHP in mixture	RT‐qPCR (gut segments) of various estrogen and xenobiotics receptors	Mimics intestinal estrogenic activity	[156]
		BPA, BPF, BPS	Humoral and cellular immune response analysis	Altered immune function (promotes intestinal inflammation)	[157]
		BPA	ELISA determination of allergen‐specific antibodies and mast cells	No activation of mast cells in the intestines	[158]
		BPS	Metabolic experiments (glucose/insulin tolerance), histological analysis	Dyslipidemia, obesity, intestinal lesions and dysbiosis	[42]
	*In vitro/in vivo*	BPA	Immunofluorescence, lysozyme activity, cells isolation, antibodies, flow cytometry, cytokines measurement	Impaired immunity and increased inflammatory response	[159]
Pig	*In vitro/in vivo*	Plasticizers, pesticides, POPs, and mycotoxins	Review	EDCs bioaccumulation	[160]
	*In vivo*	BPA	Cell counting, evaluation of neurons surface	Neurodegenerative and proinflammatory	[161]
			BPA dosing in caecum, immunohistochemistry of enteric cells	Enteric neurons calcium‐binding protein increase	[162]
Rat	*In vivo*	4‐Nitrophenol	RT‐qPCR, histomorphometry and immunohistochemistry analysis	Intestinal damage related to ER and AhR signaling pathways	[163]

Interestingly, there is a wide range of *in vitro* or *in vivo* mammalian (human, rodent, pig) and fish (mostly zebrafish) models used in this context. Various EDCs have been assessed, including bisphenols, phthalates, and numerous pesticides. While some of the publications are literature reviews of previous work, the experimental papers produced during this period show great diversity both in the impact of EDCs on metabolism and in the methods used to explore it. Studies show a range of effects, including increased intestinal permeability, impaired glucose metabolism leading to MetS, impaired hormone signaling and disruption of the immune function, reflecting the many physiological processes in which the intestine is involved, and which may be deregulated by EDCs. Methods used to highlight the impact of EDCs on the intestine and its metabolism are mainly based on measurements of key actors in intestinal metabolism, including protein expression (e.g. cholesterol) or gene expression of nuclear receptors (e.g. pregnane X receptor) and enzymes involved in the metabolism of xenobiotics. It is remarkable to note that numerous studies addressed the effects of EDCs on the intestinal microbiome underlining the recent and growing interest of the microbiome in the metabolism. In fact, even if the impact of EDCs on the gut microbiome has been investigated increasingly (2 publications in 2014 to 8 in 2018 to 21 in 2022, using the search terms “(microbiome AND “endocrine disruptors”)” in PubMed®), the impact of EDCs on the organ itself remains less investigated, even if the mechanisms by which EDCs impact the intestinal metabolism have grown over the last 10 years (Table 1). Yet, this organ is preferentially exposed to these substances as it is one of the first to come into contact with EDCs during an oral exposure.

In this review, we seek to highlight the importance of studying the consequences of MDCs on the intestine, take stock of existing *in vitro* and *in vivo* intestinal models for assessing these substances and show how the use of zebrafish could enable the development of models and bioassays for investigating the expression and the effects MDCs on enzymes such as the CYP3A family, particularly sensitive to MDCs, to facilitate the assessment of chemicals on the metabolism.

## How do MDCs impact metabolism at the intestinal level?

### MDCs promote dysbiosis

The intestinal epithelium is highly versatile as it enables the absorption of various nutrients such as glucose, amino acids, and peptides as well as minerals such as calcium [27, 28, 29]. The intestine is also involved in the absorption, synthesis, secretion, storage and degradation of lipids, whether they come from the diet (triacylglycerols, cholesteryl esters, and phospholipids) or are endogenous (phospholipids and cholesterol) [30]. Moreover, intestinal epithelial cells are also involved in the steroidogenesis of various hormones. The gut is one of the main sites for the production of glucocorticoids, as has been demonstrated in rodents and humans [31]. This versatility of metabolic activities in the organ is closely linked to its bacterial flora, the composition of which plays a major role in the processing of various dietary compounds such as proteins and lipids, as has been shown in mice [32]. Thus, the microbiota is involved in maintaining energy balance but also in fatty acid oxidation and even in the proliferation of intestinal epithelial cells [33]. Modification of the gut microbiome (composition and abundance), called dysbiosis, can lead to gut inflammation and subsequent disruption of the gut epithelial barrier and alteration of the gut motility. In fact, diversity and stability of the microbiome are essential to the proper functioning of the organism, modulation caused by the environment could cause bad health outcomes. In the case of a microbiota balance loss, various bacterial endotoxins such as lipopolysaccharides and cell capsule carbohydrates may be released and promote deleterious effects to the host, e.g. altered permeability of the intestinal barrier which can encourage the penetration of pathogenic organisms, leading to an inflammatory reaction [34]. As a consequence, the flux of metabolites between the luminal compartment and the tissue compartments is dysregulated which can lead to potential systemic inflammation, insulin resistance and lipid storage along with alterations in both appetite and lipid and glucose metabolism. Taken together, these effects can lead, ultimately, to various metabolic diseases such as obesity, type 2 diabetes and MetS as has been demonstrated in mice [35, 36]. Yet, exposure to MDCs can lead to changes in the intestinal microbiota and in the production of their metabolites. Calero‐Medina *et al*. [37] showed in humans that various classes of MDCs such as BPA and analogues disrupted the microbiota leading to a dysbiosis characterized by an increased proportion of *Microbacterium* and *Alcaligenes*. Likewise, in mice, an exposure to metals such as cadmium, reduces the presence of intestinal *Bacteroidetes* and *Firmicutes*. Moreover, beyond direct disruption by the interaction of MDCs with their regulators, many cytochromes P450 (CYPs) such as the CYP3A family, could see their enzymatic activity altered by intestinal dysbiosis, leading to modify levels of endogenous substances or xenobiotics metabolized by this enzyme family [38]. The ability of MDCs to disrupt the intestinal microbiome may therefore have deleterious consequences for its metabolism.

### MDCs disrupt the intestinal microbiome‐mediated relationships with other metabolic organs

Extra‐enteric gastrointestinal functions are one of the many physiological activities in which the intestine is involved. The gut is connected to numerous organs with which it forms specific bilateral axes (e.g. enterocerebral, enterohepatic, enteropancreatic) and even trilateral interactions (e.g. enterohepatopancreatic). This ultra‐connectivity inserts the intestine into the regulation of most of the body activities. Although many of the mechanisms enabling this connectivity are mediated by neurological and hormonal signaling through the direct secretion of intestinal and axis‐organs molecular messengers, it is also mediated by the intestinal microbiota [39]. Exchanges between the intestinal microbiota and linked organs take place via the metabolites provided by the microorganisms. A genuine communication with the host organism is established which plays a part, for example, in glucose or lipid metabolism. Thus, changes in intestinal microbiome composition and activity can lead to the disruption, in addition to damage to the intestinal tract itself mentioned above (e.g. inflammation and impaired permeability), of essential axes such as the gut‐brain axis promoting neurotoxicity [40] but also other axes involved in metabolic activities which promotes metabolic diseases such as obesity or diabetes [41]. Moreover, the disruption of the microbiome could even provide information about a particular metabolic pathology and the type of contaminants that could be the cause of this disease. For example, the identification of specific bacteria and their abundance in the dysbiosis that bisphenol S (BPS) can cause in mice could be used both as a biomarker of obesity and for detecting BPS [42]. Finally, dysbiosis caused by MDCs could be responsible for a disruption of the intestinal‐genital axis which is associated with the appearance of endometriosis [43].

### MDCs also impact the intestinal metabolism in non‐microbiome‐related ways

Several other mechanisms, unrelated to microbiota, can have deleterious effects on the gastrointestinal tract (GIT). Recently, BPA, one of the most studied obesogens, was shown to be implicated in the increase of serotonin gut levels in exposed mussels and rodents [44, 45]. In fact, BPA and its analogs increase the serotonin production by enterocytes, for a process which leads to increased insulin (which favors storage processes) secretion, lipid accumulation in the liver and lipogenesis in WAT, thus, promoting the onset of metabolic diseases in human and rodents models [46]. In another example of metabolic disruption caused by bisphenols, Mu *et al*. [47] performed zebrafish exposure to BPA and BPF showing alteration of the lipid metabolism in the intestine. Exposure to emerging pollutants can also promote the synthesis of numerous proinflammatory cytokines such as tumor necrosis factor alpha (TNFα) which is one of the main effectors of inflammatory bowel disease (IBD) by modifying tight junction proteins transcription which alters intestinal permeability [48, 49].In addition, MDCs such as PCBs are also involved in a decrease of tight junctions expression which can lead to an alteration of the intestinal permeability facilitating the entry of pathogens [50]. Intestinal endocrine functions can be altered in presence of MDCs such as di‐isononyl phthalate by lowering estradiol levels in the colon in female mice [51]. These events have been shown to be transmitted to the off‐spring [52].

### Xenobiotics metabolism in the intestine

In addition to its role in the absorption of nutrients, the GIT is also involved in xenobiotic biotransformation. These compounds pass through the intestinal epithelium and are modified by a complex detoxification system to minimize their toxic effects. The regulated permeability of the intestine, ensured, in particular, by the epithelial tight junctions [53], forms the first physical barrier met by exogenous compounds [54]. A second barrier includes a chemical dimension mediated by radical enzymes derived from the microbiome [55], which are able to carry out complex chemical transformations including hydrolysis, lyase reactions, reductive transformations, and functional group transfer reactions [32].

Numerous CYPs are expressed in the intestine, mostly localized in the small intestinal mucosa at the epithelial level [56]. Despite strong inter‐individual variations in their expressions, the CYP3A family and CYP2C9 are the most abundant enzymes expressed in the human intestine (80% and 14% respectively when immune‐quantified). CYP3A4 is the most highly expressed member of the CYP3A family [57]. These enzymes act during phase I of metabolism by modifying the redox potential of xenobiotics through mono‐oxygenation (involving O_2_) or, more rarely, through reduction. These alterations allow their elimination following conjugation by phase II metabolic enzymes e.g. UDP‐glucuronosyltransferase 1A7 allowing glucuronidation in the first‐pass intestinal metabolism, *N*‐acetyltransferase 2 allowing *N*‐acetylation as detoxification step in the intestinal epithelium or glutathione S‐transferases, essential for the metabolism of xenobiotics and protection against reactive oxygen species [58]. CYPs are also responsible for the oxidative metabolism of endogenous molecules (steroid hormones, fatty acids and vitamin D, for example). The enzymatic activities are often transcriptionally regulated by signaling pathways mediated by xenobiotic receptor/transcription factors, such as PXR (pregnane X receptor) or AhR (aryl hydrocarbon receptor), the activation of which depends upon the binding to a ligand [59].

The diversity of mechanisms that lead to intestinal disorders which can cause broader metabolic dysfunction following exposure to MDCs should be noted. Without being able to assess each of these mechanisms independently, it is interesting to focus on molecular actors capable of signaling a metabolic disorder following exposure to MDCs at the level of the intestine. To this end, the investigation of various enzymes such as CYPs, involved both in the metabolism of MDCs or, more broadly, xenobiotics, and in endogenous metabolism can provide information about the metabolic disruption caused by exposure to pollutants and offers interesting research opportunities for the years to come.

## CYP3A4, a central metabolic enzyme expressed in the intestine

In humans, the CYP3A family belongs to the CYPs group of enzymes that plays a role in the endogenous metabolism (e.g. metabolism of cholesterol by CYP3A4). This family also is involved in exogenous metabolism (e.g. pesticides and drugs) and biotransforms almost 50% of the drugs on the market [60]. Known as a major representative of the CYP3A family, CYP3A4 is present mainly in the liver and in the intestine [61]. van Waterschoot *et al*. [62] have demonstrated the importance of this CYP at the intestinal level. It acts as a protective factor for the liver in a tissue‐specific transgenic mice model that expresses human CYP3A4 either in the liver or the intestine. Through its detoxification capacities, CYP3A4 acts like an important barrier and limits systemic exposure to orally absorbed xenobiotics thus demonstrating the strong link between the two organs and the fundamental protective role played by the intestine [63].

While CYP3A4 is essential for the metabolism of endogenous and exogenous substances in humans, it is interesting to note that orthologs exist in many model species. In Table 2, we present several orthologs of human CYP3A4 in several *in vivo* models.

**Table 2:** Orthologues of human CYP3A4 in various model species.

Species	Orthologues	Localization	References
Human	CYP3A4	Hepatic/intestinal	[61]
Pig	CYP3A46	Hepatic/intestinal	[164]
*Cynomolgus* monkey	CYP3A4	Hepatic/intestinal	[165, 166]
Rat	CYP3A9	Hepatic/intestinal	[167]
Mouse	CYP3A11	Hepatic/intestinal	[168]
Zebrafish	CYP3A65	Mostly intestinal in larvae/hepatic and intestinal in adults	[169, 170]

### CYP3A4 regulators

Although CYP3A4 is regulated mainly by PXR, other transcription factors also regulate the expression of this enzyme.

#### Constitutive androstane receptor

The constitutive androstane receptor (CAR) is involved not only in xenobiotic purification processes and drug metabolism but also in lipid and glucose metabolism in the liver. CAR, thus, plays an essential role in energy homeostasis and it plays a protective role against obesity and diabetes under normal physiological conditions [64, 65]. Human CAR also regulates CYP3A4 expression, as shown in the hepatocyte cell line, HepG2 [66]. In fact, CAR appears to transactivate CYP3A4 by binding to a PXR‐binding site as shown with luminescent luciferase reporter genes in CV‐1 kidney cells (monkey) [67]. Finally, regulation of CYP3A4 by CAR can also be observed in the intestine, as demonstrated in a human colon cell line (LS174T) [68, 69].

#### Hepatocyte nuclear factor 4 alpha

The hepatocyte nuclear factor 4 alpha (HNF4α) regulates hepatic CYP3A4 expression, thus allowing ontogeny of the liver and lipid homeostasis in humans [70, 71]. Nowadays, it is well known that this nuclear receptor also is expressed in other tissues such as the intestine, where it regulates differentiation, maturation, regeneration, and cell renewal in mammals (mainly in enterocytes) [72]. This explains why deregulation of HNF4α can lead to serious intestinal pathologies associated with inflammatory bowel diseases [72]. HNF4α also plays an essential role as a direct transactivator of several CYPs involved in xenobiotic metabolism, such as CYP2C9 and CYP2C19 [73]. As an indirect transactivator, HNF4α promotes the up‐regulation of CYP3A4 by PXR and CAR in the liver in response to xenobiotics by binding to specific distal response elements upstream of the PXR and CAR response elements which are located in the proximal promoter of *cyp3a4* [74].

#### Glucocorticoid receptor

The glucocorticoid receptor (GR) is a ligand‐inducible transcription factor that regulates stress, metabolism, development and reproduction [75]. The involvement of the GR in the regulation of CYP3A4 has been demonstrated in the HepG2 human cell line [76]. Dexamethasone (DEX), a GR ligand, also has been shown to induce the intestinal expression of human CYP3A4 (and of its rat ortholog, CYP3A9, in primary cell cultures) [77]. The same study showed that DEX also induces the expression of PXR, result which supports the work of Cooper *et al*. [78] who demonstrated that GR positively regulates the expression of PXR thus promoting CYP3A4 induction in HepG2 cells.

#### Aryl hydrocarbon receptor

The aryl hydrocarbon receptor (AhR), initially known as a transcription factor linked to the toxicity of various xenobiotic compounds such as TCDD [79], also may be responsible for metabolic disorders linked to MDCs. Indirectly, it can alter the expression of PPARγ (peroxisome proliferator‐activated receptor gamma) and, thus, disrupt adipogenesis and ultimately lead to obesity [80]. When exposed to PCBs, AhR can promote an increase in *de novo* lipogenesis and a decrease in mitochondrial fatty acid oxidation. These effects facilitate the accumulation of lipids in the liver, which is the first stage in the development of hepatic steatosis [81]. In addition, high‐fat diet fed mice exposed to TCDD show worsening steatosis and liver fibrosis [20]. Among its other functions, the AhR has been shown to be involved in the regulation of various physiological processes as demonstrated by AhR‐KO mice, which display abnormalities in the liver, the GIT and in vascular development [82], by invertebrate (drosophila) and vertebrate (mice) models [83], which exhibit effects in nervous system development and by AhR‐KO mice [84], which show involvement in the immunology of barrier organs such as the skin, the lung and the intestine. Also, AhR could be involved in the regulation of CYP3A4 expression through a crosstalk with PXR, as has been suggested by studies employing a permanent hepatocyte cell line (HepaRG) and primary human hepatocytes [85]. Thus, over the last decade, the AhR has gone beyond its status as a simple purifier of xenobiotics and new functions are now being attributed to it. Indeed, this receptor appears to be involved in multiple physiological metabolic reactions and to bind several endogenous ligands (indole derivatives or tryptophan metabolites, for example). Furthermore, the high degree of conservation of the AhR across species, its constitutive expression during development and the phenotypic alterations in the AhR‐KO mice demonstrate the important role that this transcription factor plays in the physiological stability of the organism.

#### Pregnane X receptor

The nuclear receptor pregnane X receptor (PXR) was initially identified as a regulator of the expression of genes associated with the metabolism of xenobiotics. It is now known for its involvement in lipid metabolism and the maintenance of the intestinal barrier, its numerous cross interactions with other transcription factors (see below), its involvement in the metabolism and distribution of bile acids and cholesterol and in the development of hepatic steatosis [86, 87, 88]. Ubiquitous in the body, PXR is particularly abundant in the liver and the intestine [89]. It acts as an important regulator of the gut‐liver axis, both in its interaction with xenobiotics and the regulation of bile acid homeostasis genes [90]. In fact, PXR is well known for its involvement in the response to xenobiotics, the activation of PXR induces the expression of genes encoding xenobiotic metabolism enzymes such as CYP3A4, for which it is a major regulator. PXR is transactivated by a specific ligand binding to its ligand‐binding domain (LBD). This enables it to form a heterodimer with RXR (retinoid X receptor) before binding to PXR response elements (PXRE) and regulating CYP3A4 expression [91]. PXR is also involved in the expression of certain conjugation enzymes (e.g. sulfotransferase) and certain transporters (e.g. P‐glycoprotein) [92]. It is, therefore, involved in phases I, II, and III of xenobiotic metabolism and it plays a major protective role. PXR is renowned for its involvement in the metabolism of multiple xenobiotics, including drugs such as tamoxifen (anticancer agent), nifedipine (anti‐hypertensive), clotrimazole (antifungal) or other EDCs such as phthalates [78]. However, PXR also interacts with certain endogenous ligands such as lithocholic acid (steroid) and certain products of the microbial flora [93]. Thus, it appears that PXR regulates the body's homeostasis through diverse mechanisms such as the secretion of bile acids [94] and cholesterol [87], in addition to its protective role against the toxicity of exogenous substances. MDCs could therefore deregulate PXR‐mediated signaling pathways at intestinal level, leading to metabolic pathology such as hypercholesterolemia [95].

##### Interactions of PXR with other transcription factors

The human PXR receptor crosstalks with other transcription factors to regulate the expression of genes involved in the metabolism of lipids, glucose, cholesterol, bile acids and xenobiotics. For example, it interacts with HNF4a to regulate cholesterol, with the FOXO1 (forkhead box protein O1) transcription factor to regulate glucose levels and with PGC‐1α, a coactivator of the PPARγ receptor involved in gluconeogenesis [87, 96]. PXR also appears to crosstalk with the AhR receptor for regulating CYP3A4 gene expression, as it has been found in a liver human cell line (HepaRG) and primary human hepatocytes [85], thus echoing previous research in mammals [97].

## *In vitro* and *in vivo* intestinal models, to assess the impact of MDCs on the intestine

The development of bioassays and tests to evaluate the potential disruption of the endocrine system by diverse molecules is a major challenge for current research. Several experimental models have surfaced over the years. While *in silico* models are becoming increasingly important, *in vitro* and *in vivo* models still play an essential role in MDCs assessment. On the one hand, *in vitro* models are used to explore the mechanistic aspects of EDCs at the cellular level and, on the other hand, integrative *in vivo* models allow the evaluation of the potential disruption at the organismal level. Given the lack of studies on the impact of MDCs in the intestine, the development of suitable models for the evaluation of these effects in the intestine is a challenge for the future. For intestinal research, a variety of models are used today, each with its own advantages and disadvantages when compared to the human intestine. We present here an overview of currently used intestinal *in vitro* and *in vivo* models (Tables 2 and 3).

**Table 3:** Examples of *in vitro* intestinal models.

	*In vitro* intestinal models	References
Human	Caco‐2 cell line Enterocyte model isolated from a human colorectal adenocarcinoma	[171]
	HT‐29 cell line Derived from a colon adenocarcinoma, used in bioavailability and cell mechanism studies	
	T84 cell line Used to study the hormonal control of human colon carcinoma cell growth	
	Organoids – isolated crypt or stem cells to study differentiation and early immune response. 3D cell line culture to study regional absorption mechanisms and drug transport	[172]
Rodent	*In situ* intestinal perfusion Drug absorption profiling and mechanistic approaches for the absorption process (more precise than transgenic models)	[173]
Fish	RTgutGC, the first fish intestinal epithelial cell line (RT: rainbow trout), later used as epithelial barrier model RTgutF, the first rainbow trout intestinal fibroblast cell line. Combination of RTgutGC and RTgutF provides new fish intestinal barrier model New rainbow trout intestinal *in vitro* platform	[103, 104, 174, 175, 176]
	ECGI‐21, new grouper intestinal cell line to study viral pathogenesis and gastrointestinal pathologies	[177]
	No zebrafish *in vitro* intestinal model has been developed yet	

### The use of *in vitro* models

Numerous *in vitro* human intestinal models exist. For example, the human Caco‐2 cell line is a useful model since it can employ either undifferentiated cells or a differentiated monolayer that mimics the intestinal barrier. In their undifferentiated proliferating state, Caco‐2 cells exhibit a very flexible metabolism which allows fine characterization of the potentially deleterious metabolic changes that may occur upon exposure to MDCs. At confluence, Caco‐2 cells spontaneously differentiate, after 21 days, into a monolayer of polarized cells that express some specific morphological and functional properties of enterocytes [98]. This model can be used, thus, to assess the transport functions of intestinal cells and to study the intestinal barrier (morphology, biomarkers and permeability, in particular) [99], the integrity and function of which may be altered upon exposure to MDCs. The first intestinal organoid, derived from human adult intestinal crypt stem cells, was developed in 2009 following the development of gastric organoids and just preceding the development of prostate, lung and cerebral organoids [100]. Intestinal organoids also can be derived from induced pluripotent stem cells. A further degree of complexity can be added by introducing microbiota, a crucial component of the colonic microenvironment, through the injection of bacteria into the organoid lumen [101]. Finally, more complex organoids can be derived from intestinal tissue obtained following surgical resections. These tissue fragments, cultured on an air‐liquid interface, give rise to organoids containing not only epithelial cells, but also fibroblasts, immune cells, muscle fibers and cells that compose the nervous‐enteric system. This model thus recapitulates the entire microenvironment required for organoid functionality [102].

*In vitro* animal models also have evolved. Although mammalian models are now widely used, intestinal fish models are still rare. The first intestinal cell line derived from the rainbow trout (*Oncorhynchus mykiss*) appeared only in 2011 [103] and efficient epithelial barrier model only appeared much later in 2017 [104]. No *in vitro* intestinal model is currently available for the zebrafish. The characteristics of various *in vitro* intestinal models are given in Table 3.

### The use of *in vivo* models

Despite increasingly stringent regulations (mostly due to ethical considerations) and concerns about the transposability of potential adverse effects to humans, animal models still play important roles both for the investigation of the pharmacology and toxicity of substances as well as for the comprehension of the etiologies and development of pathologies in humans. In addition, the use of animal models to study endocrine and metabolic pathologies, such as obesity and its co‐morbidities, is particularly widespread and many species are used as effective tools to understand these diseases [105]. For example, studies involving rodents has allowed the identification of the role of adipose tissue inflammation in the etiology of type 2 diabetes [106]. *Caenorhabditis elegans* was used to discover the beneficial effects of flavonoids on triglyceride levels [107] and zebrafish often have been used to study hyperglycemia or hypertriglyceridemia [108]. Moreover, studies of the effects of MDCs have employed a large diversity of species as compared to conventional toxicological studies in which rodents predominate [109]. The use of species other than rodents, over and above the specific scientific interest that each particular species may present (genetic manipulation, imaging and other tools), also can be justified by the increasingly relative relevance of rodents for the study of EDCs. Despite the obvious usefulness of rodent models (as mammalian species) to mimic the effects of substances that would be observed in humans, recent research has called into question their relevance for the study of EDCs. Habert *et al*. [110] showed that there are major differences in the impact of BPA on steroidogenesis between the fetal testes of rodents and humans and even between rats and mice. In Table 4, the characteristics of various *in vivo* intestinal models are given which indicate to what extent human gastrointestinal pathophysiology can be approximated.

**Table 4:** Examples of *in vivo* intestinal models.

	*In vivo* intestinal models	References
Human	hPSC‐derived human intestinal organoids (HIOs) transplantation in immunocompromised mice showing the key features of human intestinal metabolism	[178]
Rat	Correlated to human permeability and gastric emptying rate of liquids, ideal for studying intestinal drug absorption	[178]
	Differences in the expression of metabolism enzymes such as CYP3A4, rat ortholog CYP3A9 being 11‐fold higher in the duodenum and 193‐fold higher in the colon than in humans, difference in microbiota composition	[62, 167]
Mouse	Characteristics close to the rat model, however information on the enzymes involved in metabolism is limited mainly to CYPs and ABC transporters	[178]
Pig	Greater anatomical and genetic proximity to humans which results in better replication of human gastrointestinal physiology and diseases	[179]
Zebrafish	See dedicated table below (Table 5)	

## The zebrafish as a model of growing importance in toxicology

Fish represented one quarter of the animal models used in the European Union in 2019 [105] and their use continues to increase. Among the fish, a little tropical cyprinid from the rivers of India and Malaysia, *Danio rerio* (zebrafish), is extensively used. This species is well known for its genetic proximity to humans since 70% of human genes have homologs in zebrafish [111]. Moreover, 82% of the human genes associated with the development of pathologies are related to *D. rerio* orthologs [112]. Zebrafish embryos develop, in less than a week, most of the major organ systems present in mammals, including the cardiovascular, nervous, and digestive systems. Its zootechnical properties make it an easy model to use in laboratory investigations. Zebrafish are small, they have a short reproductive cycle, fast growth and they rapidly attain sexual maturity. Further, embryonic development occurs in just a few days. More importantly, the zebrafish is a complete model that allows the exploration of the *in vivo* toxicological effects of substances on an entire vertebrate organism. It is less expensive than rodents and it is progressively becoming a reference model. The increasing use of zebrafish for pharmacological and human toxicological studies since the 1990s shows the particular interest of the scientific community for this little fish [113]. The zebrafish is employed more and more frequently as a replacement for rodent models. It provides the 3Rs (Replacement, Refinement, Reduction) value to studies, particularly at the embryonic stage (before 5 days post‐fertilization). At this stage, it is not considered an animal by law and, thus, represents an alternative model to animal experimentation [111]. The zebrafish is positioned as “a flexible model which fits between *in vitro* models and mammalian rodent models of toxicity” [114]. In addition to its zootechnical properties, *D. rerio* is an excellent model for the study of the response of specific genes to pollutants. Transgenic organisms which have fluorescent markers (e.g. Green Fluorescent Protein, GFP) linked to the expression of genes of interest are easily obtained [115]. Finally, mutants also can be easily developed to facilitate *in vivo* experimentation. For example, transparent zebrafish, named Casper, facilitate observation of the whole organism and allow fluorescence measurement from the embryonic stages to the adult [116]. *Danio rerio* is, thus, becoming an increasingly relevant model to explore endocrine disruption and its metabolic consequences in humans, particularly through the use of transgenic animals as a screening tool [108, 117].

### The zebrafish intestine, a model close to humans

**Fig. 1:**
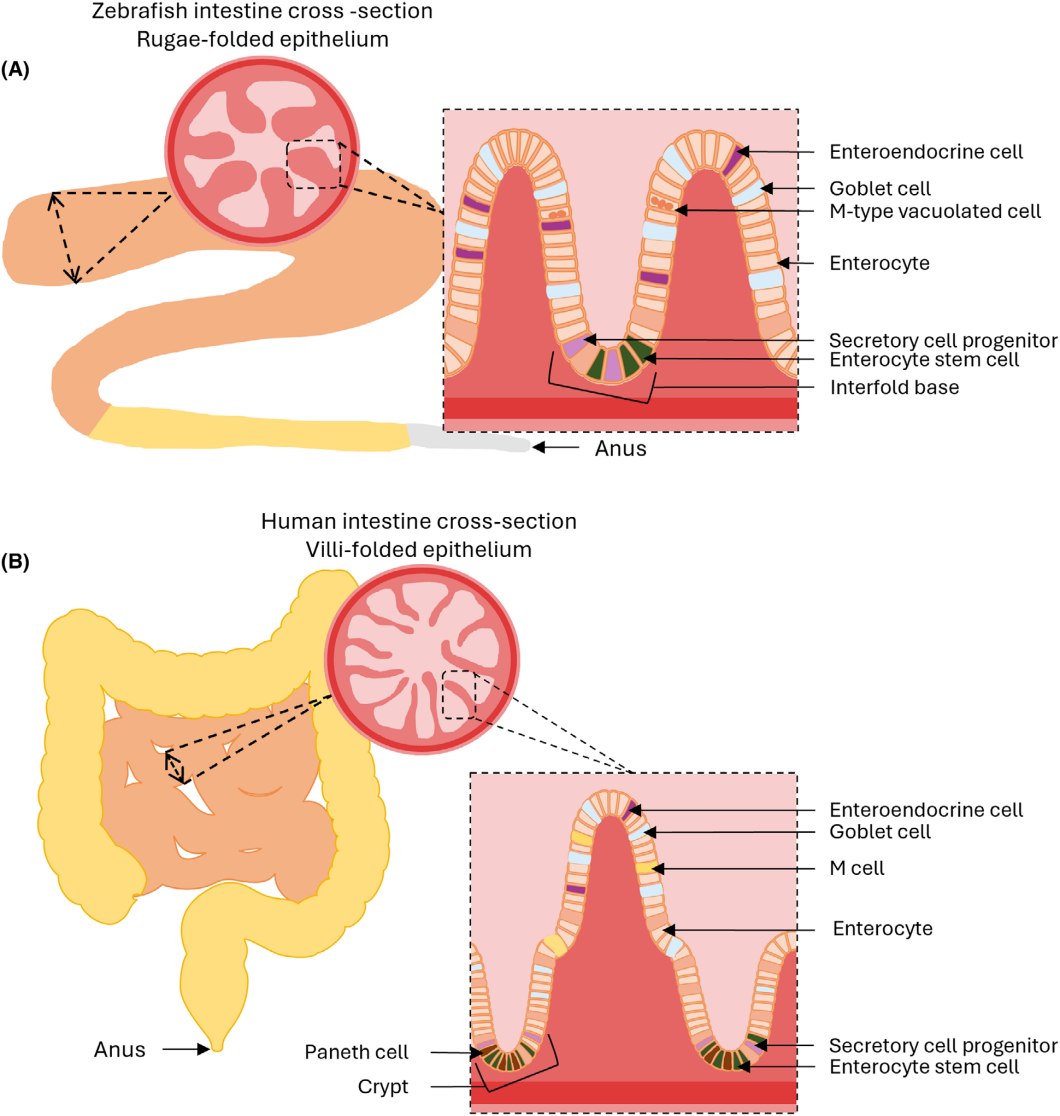
Comparison of the anatomical segments and cellular compositions of zebrafish (A) and human (B) intestines. Intestinal cross‐sections show the structural differences between zebrafish rugae and human villi. Histological sections show the main cell types for both species.

The formation of the endoderm during gastrulation in xenopus, mouse and zebrafish embryos exhibit similarities in gene expression and cellular mechanisms that allow the development of the GIT. However, the signaling pathways involved in the development of the mesoderm differ from one species to another, mainly due to differences in the regulation of the Nodal genes [118]. Thus, although the development of embryonic structures is common to all vertebrates early on, the appearance of the mesoderm marks the first difference with respect to the signaling pathways involved. Once developed, the GIT in zebrafish is different from that in mammals as it displays no stomach. Its digestive tract is composed of the mouth, the esophagus, the intestine, which is divided into three distinct parts (anterior intestine, middle intestine and posterior intestine) and the anus [119, 120]. Despite these anatomical differences, numerous studies have shown similarities in the development and structure of the intestine between mammals and zebrafish. The intestinal epithelium, for example, is particularly well‐preserved throughout evolution, performing the primitive functions of nutrient assimilation and microbiota interaction [121, 122, 123]. The intestinal epithelial cells can be divided into two categories, depending upon whether they play an absorptive or secretory role. In humans, the enterocytes (M cells and BEST4+ cells) are involved in absorption whereas enteroendocrine, tuft, goblet and Paneth cells are involved in the secretion of diverse compounds including mucus [124]. Intestinal cells are arranged in villi in humans and zebrafish have a comparable folded structure, called rugae. Human villi form crypts which contain intestinal stem cells that migrate along the villi during differentiation and Paneth cells. Zebrafish rugae do not form crypts but rather interfold bases containing progenitors of enterocytes and secretory cells. The apical domain contains differentiated cells in both humans and zebrafish. Although the histological organization is slightly different (unlike mammals, zebrafish do not have a submucosal layer and the smooth muscle layer is simpler and directly attached to the mucosa), the zebrafish intestinal epithelium is broadly similar to that of mammals, with a specialization of epithelial cells to ensure the various physiological functions of the intestine [125, 126]. Zebrafish present the same heterogeneity in cellular specialization as attested to by single‐cell RNA sequencing. However, they do not have Paneth and M cells, both of which are involved in intestinal immunity, although a vacuolated M cell type is recognizable which performs the mammalian‐like functions in passive immunity [125, 127]. Similarities in the structures and cellular compositions, at the anatomical and cellular level, of the zebrafish and human intestines are illustrated in Fig. 1.

The specialization of the epithelial cells is reflected by specific gene expression in the different zones along the intestine. These genes have been identified in several mammalian and fish species (human, mouse, zebrafish, and stickleback) [128]. When the zebrafish intestine is artificially divided into seven equal parts on the basis of specific gene markers, the epithelial cells show a large number of orthologous genes between zebrafish and mice. There is a strong correlation in the expression of specific markers between the two species in the first 5 segments (equivalent to the small intestine in mammals) [129]. Thus, some equivalences can be identified. The zebrafish anterior intestine is similar to the duodenum and jejunum of mammals as based on the *ada* and *rbp2a* gene markers, respectively, and it shares similar functions (absorption of lipids, carbohydrates, and proteins). The zebrafish middle and posterior intestines are similar to the mammalian ileum and colon, as based on the *fabp6* and *lamp2* marker genes, respectively, and they share similar functions. The middle segment absorbs bile salts, like the ileum, whereas the posterior segment absorbs ions and water, similarly to the colon [126, 128, 130].

### Zebrafish intestinal *in vivo* models

Several zebrafish intestinal models are available which can serve as tools for the study of the intestine from diverse perspectives, including toxicology. Here, we present the models currently used to investigate the intestine and its specific features (Table 5). Mutant and transgenic zebrafish models can be used to assess the toxicity of substances on the GIT, to study (a) the inflammation of the intestinal tissue, including in chronic diseases such as enterocolitis and IBD, and (b) the development of intestinal cancers and potential target therapies. Of the various zebrafish intestinal models, larval models are frequently used as they offer several advantages. In addition to the large number of individuals generated at this stage, the larvae can be manipulated easily. Moreover, live imaging is easy, as is the colonization of the digestive tract by specific bacteria. Finally, high‐throughput drug screening is possible at this stage.

**Table 5:** *In vivo* intestinal zebrafish models currently used in experimental research.

Models	Relevance to humans	Specificities	References
I‐FABP GFP/RFP‐transgenic zebrafish	Intestinal‐type fatty acid‐binding protein (I‐FABP) is implicated in fatty acid trafficking and metabolism in gut and is present in both zebrafish and mammals	GFP and RFP fluorescent reporters reflect the expression of I‐FABP in the developing and mature gut. Could be used as an *in vivo* model to assess functional analysis of the intestine	[180]
Tg(cyp2n13p:egfp) larval model	Orthologue of human cyp2j2, involved in liver xenobiotic metabolism (including antihistamine drugs)	Four transgenic models for assessing drug‐induced organ toxicity at different phases of drug metabolism enabling *in vivo* screening of substances, particularly useful during drug development. While these biomarkers are not exclusive to the GIT, since they are also present in the liver, their expression is much more pronounced at the intestinal level, underlining the primary role of the gut in oral substance metabolism	[181]
Tg(gsr:egfp) larval model	Glutathione s‐reductase (gsr) involved in xenobiotic metabolism		
Tg(gstt1b:egfp) larval model	Glutathione s‐transferase theta 1b (gstt1b) endobiotic metabolism		
Tg(cyp2k18:egfp) larval model	Orthologue of human cyp2w1, marker of colon cancer and hepatocellular carcinoma		[182]
Microbiome‐free ZF larvae fed on microbe‐free live food rearing protocol	Depending on which bacteria with which the model is infected	Allows the study of interactions between host commensal and pathogenic bacteria by controlling zebrafish larvae microbiome composition	[183]
Mutant (tp53^M214K^) in *Helicobacter pylori* CagA oncoprotein context	Orthologue mutation of methionine 246 missense mutations identified in human tumors	p53 loss is sufficient to induce high rates of adenocarcinoma and small cell carcinoma in the zebrafish intestine	[184]
APC‐mutants larval model	Mimics the mutations found in Familial Adenomatous Polyposis (FAP) patients	Premature stop codon leading to digestive tract neoplasia	[185]
Tumor cells transplantation using *rag2* ^*E450fs*^ mutants	Relevant to human biology, comparable to xenograft mouse models	A xenograft model enabling visualization of cancer development, not specific to gastrointestinal oncology	[186] [187]
Tg(ifabp:EGFP‐kras^V12^)	Mimics intestinal tumor formation in humans	A transgenic model detecting overexpression of k‐Ras^V12^, an oncogenic factor in the intestine which promotes intestinal carcinogenesis, enteritis, epithelial hyperplasia, and tubular adenoma in adult fish	[188]
Larval and adult models for enterocolitis and IBD etiology	The similarities observed between the intestinal physiology of humans and zebrafish are also present in the pathological context, particularly in enterocolitis and IBD	A relevant model to study intestinal inflammation through the expression of proinflammatory marker as TNFα, leukocytosis, bacterial overgrowth, alteration of goblet cells, epithelial disruption, etc. pathologies are induced by exposure to substances (TNBS for example)	[125]
APOA‐I‐mCherry transgenic zebrafish larval model	First evidence of cholesterol transport in the intestinal endosomal‐lysosomal trafficking system. Model is relevant to humans due to the difficulty of imaging the transport of nutrients and proteins in vertebrates and the similarities between zebrafish and human intestine	A novel assay for imaging apolipoprotein A‐I and live dietary cholesterol trafficking in the zebrafish intestine by feeding transgenic larvae expressing an APOA‐I fluorescent fusion protein (APOA‐I‐mCherry) with TopFluor‐cholesterol, a fluorescent cholesterol analog	[189]

#### Current approaches

Recently, several large‐scale European projects have led to the creation of bioassays which use zebrafish models. For example, the European OBERON project, which was launched in 2019, aims to obtain “an integrative strategy of testing systems for identification of EDCs related to metabolic disorders” [131]. In this context focusing on human health, a number of *in vivo* zebrafish models have been developed. Considering the proximities between zebrafish and humans, and the existence of a Steatogenic Assay on Zebrafish (StAZ), a new bioassay using larvae from a zebrafish line expressing a blue, fluorescent liver protein reporter to screen and characterize steatogenic EDCs [132] has been developed. The Zebrafish Obesogenic Test (ZOT) was also used in this project. This test assesses the effects of substances on WAT in zebrafish larvae, in order to ascertain the obesogen potential of an EDC [133].

The zebrafish at the embryonic stage, thus, allows the creation of models and bioassays alternative to animal models that are useful for assessing the impact of EDCs on metabolic diseases. It is, therefore, realistic to imagine the development of similar bioassays for the intestinal. Models developed for other organs (liver or adipose tissue) rely on precise molecular targets, such as a hepatic protein in the StAZ bioassay and specific intestinal targets also can be envisioned for the investigation of deregulations of gut metabolism.

## The zebrafish CYP3A65 gene: a potential marker of intestinal disturbance in the context of MDCs?

As an orthologue of human CYP3A4, highly expressed in the intestine and extensively involved in gut metabolism, CYP3A65 could represent a first‐rate molecular target for the development of a relevant intestinal bioassay. This cytochrome is mainly expressed in the anterior part of the zebrafish intestine (Fig. 1) [134]. The *cyp3a65* gene has 54% similarity to human *cyp3a4* [135]. Its expression seems to be regulated by both zfPXR and zfAhR, both of which are orthologs of the human transcription factors PXR and AhR according to the limited literature available on this cytochrome. In fact, Chang *et al*. [136] suggest that zfAhR and zfPXR are both necessary for *cyp3a65* expression, consistently with Jackson and Kennedy [137] who advance that zfPXR plays a role in its expression and Salanga *et al*. work which point out that although zfPXR is involved in CYP3A65 expression, the depletion of this transcription factor does not lead to the silencing of the cytochrome expression which suggests a compensatory mechanism [138]. Finally, Kubota *et al*. [139], showed that the expression of CYP3A65 is regulated by both zfAhR and zfPXR through an interaction resembling reciprocal crosstalk between the two transcription factors. These results recall the complex regulation of human CYP3A4 described above. Overall, research to date has led to different hypotheses for the regulation of CYP3A65, involving zfAhR and/or zfPXR, thus calling for further investigation into the expression of this cytochrome.

### Factors regulating CYP3A65 expression: comparison with humans

Recent studies in zebrafish have demonstrated that CYP3A65 is strongly regulated by zfPXR and zfAhR, in humans, other transcription factors such as CAR, HNF4α and GR have been shown to regulate CYP3A4 expression (see dedicated section above). CAR is considered “lost” in teleost fish such as zebrafish [140], whereas PXR shows strong interspecies differences. In fact, in addition to its large number of ligands, PXR displays some interesting species‐specificity. For example, zebrafish PXR (zfPXR) has a smaller LBD pocket [141]. This could explain why the same chemical may have different effects on PXR activity following binding to zebrafish or human PXR (hPXR) [142]. Similarly, the pharmacophore analysis of PXR in several species (mouse, rat, chicken, zebrafish, human) shows strong differences. For example, the same molecule (Benzo[a]pyrene) is active in some species (zebrafish, rat, humans) and inactive in others (rabbit) [141]. *In fine*, although some species are closer to humans, no perfect model exists, all differ from humans to a greater or lesser extent in terms of PXR activity.

The study of the expression of zebrafish CYP3A65 for the investigation of exposure to MDCs could provide information on the impact of these substances on human CYP3A4. For example, following the logic of the intestinal transgenic embryonic models already in use, we suggest that a model that allows the measure of CYP3A65 expression by GFP fluorescence, following exposure to a substance, could be used to screen a wide range of molecules and their effect on CYP3A65 expression. This model could be employed, also, to investigate possible crosstalk between the AhR and PXR signaling pathways, which could indicate possible effects of these substances on human intestinal CYP3A4 expression.

## Conclusion

The role of MDCs in the development of metabolic diseases in humans has stimulated important research efforts which range from mechanistic to epidemiology studies. In this review, the pleiotropic role of the intestine and its importance in numerous physiological functions, from its role as a barrier and endocrine organ to its role in the metabolism of xenobiotics and endogenous compounds are underlined.

Intestinal CYPs represent relevant molecular and biochemical targets for investigation on the effects of MDCs as they are highly expressed, they are involved in the metabolism of endogenous compounds, and they are targeted by numerous pharmaceuticals.

This review highlights the CYP3A family, among the CYPs, as being particularly relevant because of its level of expression and the number of chemicals that could potentially disrupt CYP3A expression/activity. The development of relevant biological models to explore the modes of action and the effects of MDCs at the intestinal level is of prime importance for the development of specific bioassays to determine the biological activities of key target genes and evaluate physiological processes. The zebrafish model is of particular interest in this context given the similarities of its GIT with that of humans. Several *in vivo* zebrafish intestinal models have recently emerged for the investigation of various human pathologies, such as colon cancer and IBD, as well as for the study of gut microbiota, intestinal nutrient transport and the toxic consequences of drugs on the GIT. One can expect that the development and the implementation of specific *in vivo* zebrafish models will constitute novel opportunities for the further study of the modes of action and the effects of MDCs in the intestine in concert with additional new approaches to evaluate integrated multi‐organ toxicity.

## Take‐home messages

1. The role of the intestine on the effects of MDCs, has been unexplored as compared to other organs.
2. Several CYP450s are regulated at the transcriptional level, by one or several xenobiotic receptors (e.g., the CYP3A family).
3. The zebrafish model shares numerous similarities with humans, particularly its GIT.
4. The zebrafish model represents an opportunity to study the complex interactions of MDCs at the intestinal level and the subsequent development of pathologies.
5. Expect the development of pathological intestinal zebrafish models that could be used for
  - Drug development.
  - Regulatory assessment of chemicals.
  - Fundamental research on the mode of actions of such chemicals.

## Conflict of interest

The authors declare no conflict of interest.

## Author contributions

Paper conceptualized by CE, XC and FB; research and writing by CE; paper reviewed by CE, XC, FB, SA‐A and SB.
